# Heads First: Visual Aftereffects Reveal Hierarchical Integration of Cues to Social Attention

**DOI:** 10.1371/journal.pone.0135742

**Published:** 2015-09-11

**Authors:** Sarah Cooney, Holly Dignam, Nuala Brady

**Affiliations:** 1 School of Psychology, University College Dublin, Belfield, Dublin, Ireland; 2 School of Bimolecular and Biomedical Science, University College Dublin, Belfield, Dublin, Ireland; Monash University, AUSTRALIA

## Abstract

Determining where another person is attending is an important skill for social interaction that relies on various visual cues, including the turning direction of the head and body. This study reports a novel high-level visual aftereffect that addresses the important question of how these sources of information are combined in gauging social attention. We show that adapting to images of heads turned 25° to the right or left produces a perceptual bias in judging the turning direction of subsequently presented bodies. In contrast, little to no change in the judgment of head orientation occurs after adapting to extremely oriented bodies. The unidirectional nature of the aftereffect suggests that cues from the human body signaling social attention are combined in a hierarchical fashion and is consistent with evidence from single-cell recording studies in nonhuman primates showing that information about head orientation can override information about body posture when both are visible.

## Introduction

Facilitated by a number of visual cues, such as eye-gaze, pointing gestures, head orientation and body posture, the ability to discriminate the direction of another person’s attention is an important skill in social interaction. The fast and efficient discernment of where someone else is attending permits joint attention, an ability that emerges early in human development and that is integral to the development of theory of mind [1].

Psychophysical studies that address how the various cues from the body signal the direction of one’s attention can be divided methodologically; those that employ attention orienting and interference paradigms [2–5] and those that use visual adaptation to examine the representation of eye-gaze direction, head orientation and body orientation [6–9]. Studies employing visual adaptation almost invariably examine these cues in isolation, while studies investigating the important question of how these cues are integrated typically employ the cueing and interference paradigms. Here we use a *cross-category* adaptation paradigm in what is, to our knowledge, the first study to use this method to ask how the cues of head and body orientation interact in signaling the direction of social attention. Our results provide clear evidence for a hierarchical model of cue combination initially proposed to explain single cell data [10].

Perrett and colleagues [10] propose a top down hierarchy of cues to social attention in which a Direction of Attention Director (DAD) combines cues of eye-gaze, head orientation and body posture in order to determine where another person is attending. As eye-gaze is most informative about the direction of attention in cases of cue conflict, it is placed at the top of this hierarchy and supersedes information about head orientation, which, in turn, supersedes information about body posture. The likely site of the DAD is the superior temporal sulcus (STS) where cells responsive to both gaze direction and head orientation are found. These cells respond optimally when eye-gaze and head turning are in the same direction and their responses are modulated by gaze direction in the case of conflicting cues from the head [10,11].

Experimental support for this hierarchical model of cue combination is, however, somewhat mixed. Research using the modified Posner cueing paradigm–which measures the time to shift attention—supports an integrative hierarchical model such that eye-gaze is referenced to head orientation and head orientation is referenced to body orientation [3,4]. In contrast, studies using Stroop-like tasks report a bidirectional interference effect whereby information from eye-gaze does not completely inhibit information from head orientation nor *vice versa* [5,12]. The symmetry of the interference effects are consistent with a parallel processing model, whereby head orientation and eye-gaze direction cues are processed separately, yet have additive effects on the discrimination of social attention [5].

One explanation for the discrepant findings is the task requirements, such that visual information for attention orienting (as studied in the spatial cueing paradigm) is processed differently than for overt direction discrimination (as is used in the Stoop-like task). Here we employ a visual adaptation paradigm in which participants are asked to explicitly discriminate head and body turning direction to examine how these cues are integrated by the visual system.

Adaptation is a general property of the visual system such that our perceptual experience is mediated by continuous adjustments in neural sensitivity that reflect the recent history of what we are looking at [13]. Adaptation aftereffects manifest as perceptual biases that are induced after exposure to a stimulus and that are thought to reflect a reduction in the response of cells tuned to specific features of that stimulus. Therefore, the measurement of aftereffects provides a way to explore the neural coding of various stimulus dimensions [14]. For example, exposure to lines that are tilted to the right or to the left leads to a perceptual shift such that subsequently viewed vertical lines appear to tilt in the opposite direction [15], thus revealing the operation of neural ‘channels’ tuned to orientation. Visual adaptation has been employed to explore the neural coding of ‘low level’ stimulus properties such as color, motion, orientation and curvature, as well as ‘high level’ properties such as facial identity and emotional expression, see [16,17] for reviews.

Pertinent to the current research are a number of studies that have used visual adaptation to explore the perception of body and head orientation. Lawson and colleagues [8] report a viewpoint aftereffect in the perception of body orientation such that after adapting to bodies that are oriented either to the right or left participants perceive forward facing test bodies as turned in the opposite direction. And a very similar viewpoint aftereffect is reported in the perception of human heads [9]. In both studies the pattern of aftereffects is consistent with a multichannel model in which separate mechanisms code the direction of left, right and forward facing bodies or heads. For example, adapting to an alternating sequence of heads oriented 20° to the right and to the left leads to an increased tendency to judge heads that are oriented a little to the right or left as ‘forward facing’. In contrast, adapting to forward facing heads sharpens orientation tuning, so that faces that are oriented a little to the right or left are now more likely to be correctly categorized as such. This result is consistent with a multichannel but not opponent coding model [9].

Using *cross-category* adaptation, Fang and He [6] found that the viewpoint aftereffect does not transfer between object groups. Specifically, adapting to faces oriented to the right or left did not produce a perceptual shift in judging the orientation of a selection of non-biological control stimuli including cars and wireframe objects. Perceptual aftereffects were found within all these object categories but not between categories. Although the potential of this technique to reveal shared or separate neural representations is considerable, *cross-category* adaptation has not been extensively employed to study the representation of the human body. An exception is recent research on the representation of gender, with reports that adapting to images of male or female bodies elicits an aftereffect such that the perception of faces is then biased toward the opposite gender [18,19]. Similarly, recent research on preference for facial shape shows that viewing heavy bodies enhances preferences for facial adiposity [20]. Given the considerable physical difference between heads and bodies, such ‘cross-category’ aftereffects must reflect particularly high-level representations.

The experiments reported below investigate whether adapting to images of heads (or bodies) presented in side view produces a perceptual bias in judging the direction of orientation of bodies (or heads). Experiment 1 measured participants’ perception of body orientation before and after adapting to bodies or heads which were extremely oriented to the right or left. Strong aftereffects were expected in the *same-category* condition given previous findings [8]. While previous research shows that viewpoint aftereffects do not transfer across object categories [6], *cross-category* adaptation has not been previously tested using different parts of the body. Assuming different cues to social attention are commonly coded at some level of visual processing [10] we expected that adapting to extremely oriented heads would influence the perception of body orientation. Experiment 2 measured participants’ perception of head orientation before and after adapting to heads or bodies extremely oriented to the right or left. Again, strong aftereffects were expected in the *same-category* condition. Assuming a hierarchical coding of cues to social attention such that cues from head orientation override those from body orientation processing [10], the effects of *cross-category* adaptation were expected to be weaker or absent.

## Methods

Sample size was chosen in advance of data collection, is comparable to that used in research on high level aftereffects [8,9] and is consistent with sampling in psychophysics where all participants typically show the effect [21]. Each participant’s data were checked to ensure they showed adaptation and no observations were excluded from the analyses.

### Participants

Three distinct groups of twelve (6 female, 6 male) right-handed volunteers from the UCD student population participated in Experiments 1, 2 and 2(a). All were naïve to the purpose of the experiments, had normal or corrected-normal vision and received ten euros for their participation. Mean [and SD] age was 21 [0.95] years, 27 [5.87] years and 22.6 [5.5] years for the three groups. The study was approved by the UCD Research Ethics Committee; in accordance with the Declaration of Helsinki all participants gave written, informed consent and were advised of their right to withdraw from the study at any time without prejudice.

### Stimuli

The stimuli were computer-generated images of human bodies and heads created using Poser http://poser.smithmicro.com. The bodies were cropped at the neck, just below the hips and at the wrists so as to remove orientation cues that may be provided by the hands or legs. The heads were generated without hair, were cropped at the neck and had the eyes closed so as to remove any eye-gaze cues to orientation.

**Fig 1:**
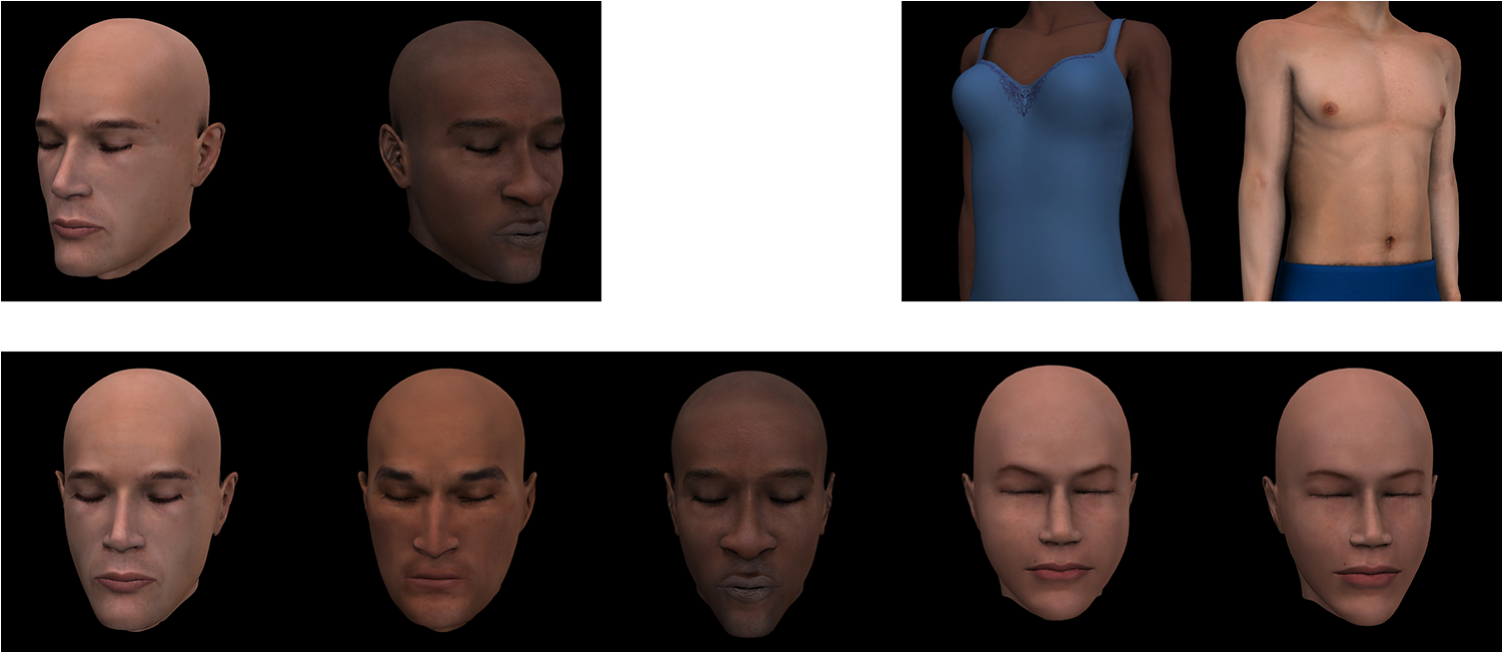
Examples of the test heads at angles of 4° and 2° left, 0°, 2° and 4° right (bottom panel) and of adapting heads and bodies oriented 25° right and left (top panel).

In Experiment 1 the test stimuli depicted bodies of 10 identities, 5 male and 5 female, each positioned at 8° left, 4° left, 0°, 8° right and 4° right. For *same-category* adaptation, the adapting stimuli were the same 10 bodies oriented 25° left and 25° right. For *cross-category* adaptation, the adapting stimuli comprised 10 different identities of heads (5 female, 5 male) with similar variation in shape and skin tone as in the bodies, each oriented at 25° left and 25° right (Fig 1).

In Experiment 2 heads (at 4° and 2° left, 0°, 2° and 4° right) served as the test stimuli, and heads (bodies) at 25° right and left served as the adapting stimuli in the *same (cross) category* variants. We chose smaller angles for the test stimuli in Experiment 2 because participants’ discrimination of head orientation was finer than their discrimination of body orientation in Experiment 1. For completion, we also present the results of a preliminary study, Experiment 2(a), as supporting information, in which heads (at 8° left, 4° left, 0°, 8° right and 4° right) served as the test stimuli, and heads (bodies) at 25° right and left served as the adapting stimuli.

Images were rendered in colour at 756 x 756 pixels and subtended, vertically, ~21.2° of visual angle at a viewing distance of 60cm. To avoid retinotopic adaptation in the *same-category* conditions adaptors were made 15% larger than the test stimuli. The stimuli were presented and participants’ responses recorded using Presentation running on a Dell XPS-8300 PC with a screen size of 19 inches and display resolution of 2048 by 1152 at 60 Hz.

### Procedure

The procedure followed that of published research [8,9]. In both the *same-category* and *cross-category* adaptation variants participants completed 3 experimental phases, a pre-adaptation (baseline) phase and an adaptation phase, which was repeated for the 25° left and 25° right adaptors, and a test phase during which participants were also exposed to top-up adaptation. To ensure no carry over adaptation effects participants had a 10-minute break between right and left adaptation phases, and completed the same and cross category variants at least 1 day apart.

In the pre-adaptation phase of Experiment 1 all 10 bodies were shown at each of the 5 orientations for 50 trials. Baseline trials began with a central fixation cross (750ms) followed by a test body (300ms). The screen was then blanked until the participant responded using the number pad keys 1, 2 and 3 to indicate whether they perceived the body orientation as “left”, “direct’ or “right” respectively. Presentation order was pseudo-randomized.

The adaptation phase started with an adaptation period of ~ 4mins where the 10 adaptation stimuli (5 male and 5 female bodies or heads, oriented 25° to the right or left) were presented 5 times each for 4000ms followed by a blank 750ms ISI. Trial order was pseudo- randomized and participants stated the gender of each stimulus to maintain attention. Adaptation orientation was counterbalanced so that half the participants adapted to leftward and half to rightward oriented stimuli first.

The adaptation was immediately followed by a block of 50 test trials during which adaptation was topped up. Each top up adaptor was presented for 6000ms followed by a test for 300ms with the word ‘RESPOND’ printed beneath it. Participants indicated whether the test bodies were oriented left, direct or right using the number pad. The procedure was identical in Experiment 2, except that heads served as the test stimuli and bodies as the adaptors.

## Results

The percentage of ‘straight ahead’ responses was analyzed in R [22] using ANOVA with within-subjects factors of stimulus *Orientation* (5 levels), *Adaptation* (3 levels), and *Condition* (*same/cross-category*). Greenhouse-Geisser corrections were used when Mauchly's Test for Sphericity was significant and effect sizes are given by generalized eta squared (*η* ^*2*^_*G*_) [23]. Following Cumming [24], significant interactions are explored by plotting and reporting point estimates and associated confidence intervals in lieu of significance testing.

### Experiment 1 Same (Body-Body) & Cross (Head-Body) Category Adaptation

**Fig 2:**
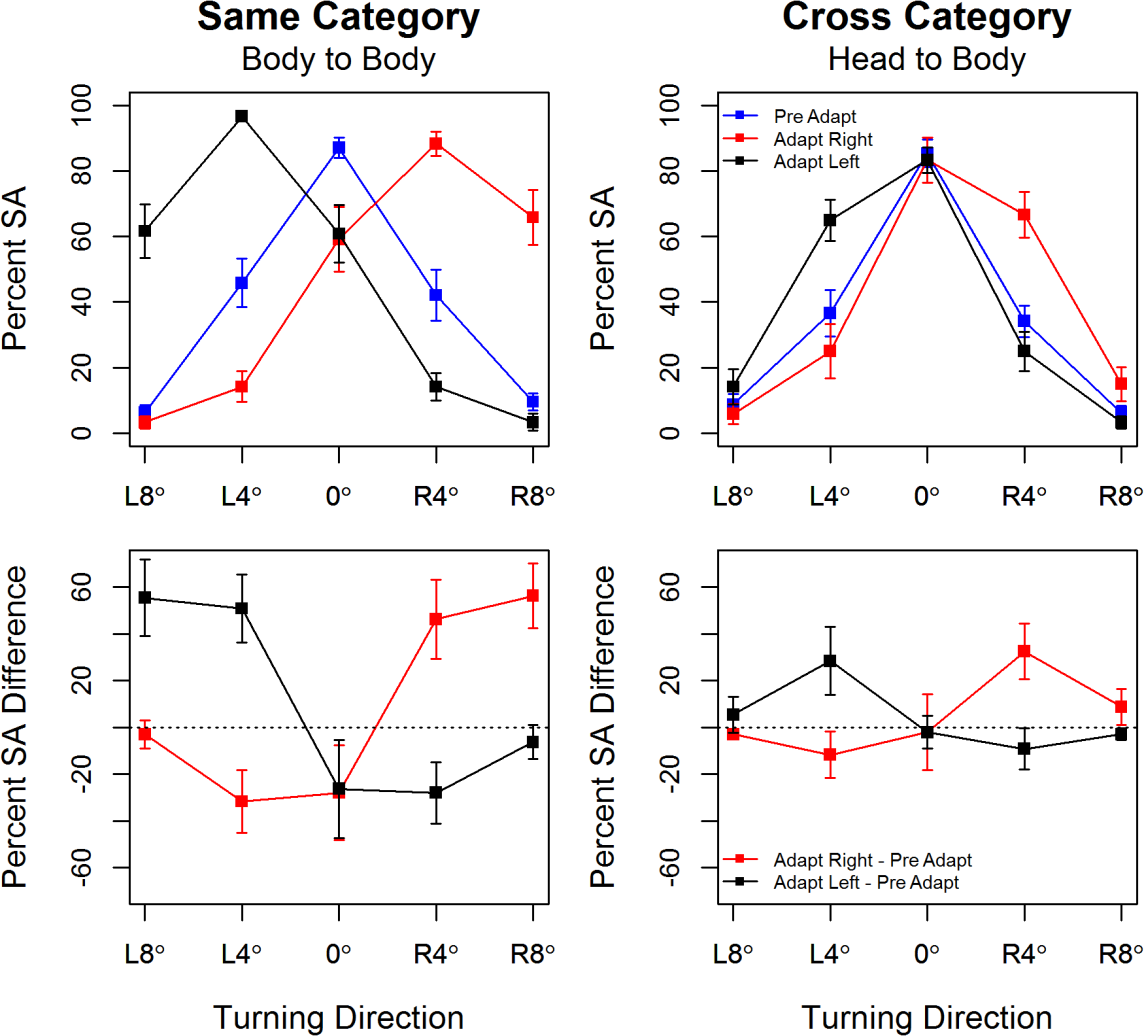
Top Panel: The percentage of straight ahead responses is plotted by test body orientation with separate traces for pre-adapt, post-adapt right and post-adapt left. Error bars show +/1 S.E.M. **Bottom Panel**: Mean difference in the percentage of straight ahead responses pre- and post-adaptation by test body orientation, with separate traces for adaptation to rightward and leftward facing adaptors. Error bars show 95% confidence intervals around the point estimates, the dotted black line marks an effect size of zero.

The percentage of ‘straight ahead’ responses is plotted by body orientation in Fig 2 where there is evidence of strong adaptation in the *same-category* condition. At baseline participants show high accuracy in judging the facing direction of bodies oriented at 0° and 8°, but often (~40% of trials) judge bodies oriented 4° left and right as facing straight ahead. After adapting to bodies orientated 25° right or leftward, the response curves shift in the direction of the adapting stimulus, so that the percentage of straight ahead responses now peaks at 4° right or left respectively. This shift in the ‘neutral point’ is characteristic of many forms of adaptation [16]. Note also that tuning is sharpened for bodies oriented in the opposite direction to the adapting stimulus, i.e., after right (*left*) adaptation participants are more likely to correctly perceive 4° left (*right)* bodies as oriented to the left (*right*). See also Table 1.

**Table 1:** Response Change Post Adaptation Compared to Baseline.

**Experiment 1** Same Adaptation (Body to Body)				
	*Adapt Right*: *Baseline*		*Adapt Left*: *Baseline*	
**Test Body**	**Response Change**	**Percent SA Change [95% CI]**	**Response Change**	**Percent SA Change[95% CI]**
8° L	↓SA, ↑L	-2.92 [-8.89:3.06]	↑SA, ↓L	55.42 [39.02:71.81]*
4° L	↓SA, ↑L	-31.67 [-45.03:-18.30]*	↑SA, ↓L	50.83 [36.25:65.41]*
0°	↓SA, ↑L	-27.92 [-48.21:-7.62]*	↓SA, ↑R	-26.25 [-47.24:-5.26]*
4° R	↑SA, ↓R	46.25 [29.32: 63.18]*	↓SA, ↑R	-27.92 [-41.08:-4.75]*
8° R	↑SA, ↓R	56.25 [42.36:70.14]*	↓SA, ↑R	-6.25 [-13.56:-1.06]
**Experiment 1** Cross Adaptation (Head to Body)				
	*Adapt Right*: *Baseline*		*Adapt Left*: *Baseline*	
**Test Body**	**Response Change**	**Percent SA Change [95% CI]**	**ResponseChange**	**Percent SA Change [95% CI]**
8° L	↓SA, ↑L	-2.92 [-5.04:-0.79]	↑SA, ↓L	5.42 [-2.18:13.01]
4° L	↓SA, ↑L	-11.67 [-21.56:-1.77]*	↑SA, ↓L	28.33 [13.79:42.88]*
0°	↓SA, ↑L	-2.08 [-18.25:14.08]	↓SA, ↑R	-2.09 [-9.05:4.88]
4° R	↑SA, ↓R	32.5 [20.72: 44.27]*	↓SA, ↑R	-9.17 [-18.03: -0.30]*
8° R	↑SA, ↓R	8.75 [1.19:16.31]	↓SA, ↑R	-2.92 [-5.44: -0.39]

Note. SA = Straight Ahead responses; L = Left responses; R = Right responses.

* Substantial effect in the expected direction, and the 95% CIs do not include zero

There is similar evidence of adaptation in the *cross-category* condition. Although with no obvious shift in the neutral point, the same characteristics of negative aftereffects outlined by [16] are present, with bodies oriented in the same direction of the adapting head stimuli now perceived as facing straight ahead. And tuning is sharpened for bodies oriented in the opposite direction to the adapting heads, i.e., after right (*left*) adaptation participants are more likely to correctly perceive 4° left (*right)* bodies as oriented to the left (*right*). See alsoTable 1.

As expected from these observations, the omnibus ANOVA test showed a significant 3-way interaction, Orientation*Adaptation*Condition, F(8,88) = 16.93, *p* ~0.00, *η2G* = 0.20, so separate analyses for the *same-* and *cross-category* adaptation conditions were run. Both ANOVAs showed significant main effects of *Orientation* and of *Adaptation* whose interpretation is qualified by significant Orientation*Adaptation interactions, F(8,88) = 50.95, *p* ~ 0.00, *η2G* = 0.66 for *same-category*, and F(8,88) = 15.79, *p* < 0.01, *η2G* = 0.28 for *cross-category* adaptation.

These interactions were explored using planned contrasts of the percentage of straight ahead responses prior to and after adapting at each of the five test body orientations. In interpreting these results we follow the ‘new statistics’ approach [24] and plot point estimates of effect sizes (the mean difference in the percentage of straight ahead responses pre- and post-adaptation) with associated 95% CIs in the lower panel of Fig 2. In Table 1 we note with an asterisk those rows in which the effect is in the expected direction, where the absolute effect size is substantial *and* where the 95% CIs do not include zero.

For *same-category* adaptation these effect sizes run between a low of 26.25% and a high of 56.25%. For *cross-category* adaptation these effect sizes run between a low of 9.17% and a high of 32.5% and occur after both rightward and leftward adaptation for test stimuli of 4°L and 4°R. Effect sizes and associated 95% CIs are reported in lieu of significance tests given the high sampling variability of p-values [24]. Cumming [25] notes that ‘only very small p-values give a reasonable basis for rejecting a null hypothesis’ (p134–135), and that in these cases effect sizes are typically large and unequivocal. We note that where our effect sizes are largest (>30%) paired t-tests all return p-values < 0.001.

To summarize, while the effect sizes are greater after same category (body to body) adaptation, there is also clear evidence of robust adaptation in the cross category (head to body) case.

#### Experiment 2 Same (Head-Head) and Cross (Body- Head) Category Adaptation

Experiment 2 measured participants’ perception of head orientation before and after adapting to heads or bodies oriented 25° to the right or left. In our preliminary study, Experiment 2(a), the test heads were presented at the same angles as the test bodies in Experiment 1, namely 8° and 4° left, 0°, 8° and 4° right. Here *same-category* adaptation was strong whereas there was no evidence of *cross-category* adaptation. (See S1 **Fig**)

This lack of *cross-category* adaptation may reflect the proposed hierarchy whereby cues to social attention from the head may override those of the body. However, the lack of adaptation may, in part, reflects participants’ finer discrimination of head orientation than of body orientation for the stimuli as rendered here, e.g., participants are making ~40% errors for bodies oriented at 4° in the baseline condition (Fig 2) whereas their baseline error rate for heads oriented at 4° is ~ 20% (**S1 Fig**).

**Fig 3:**
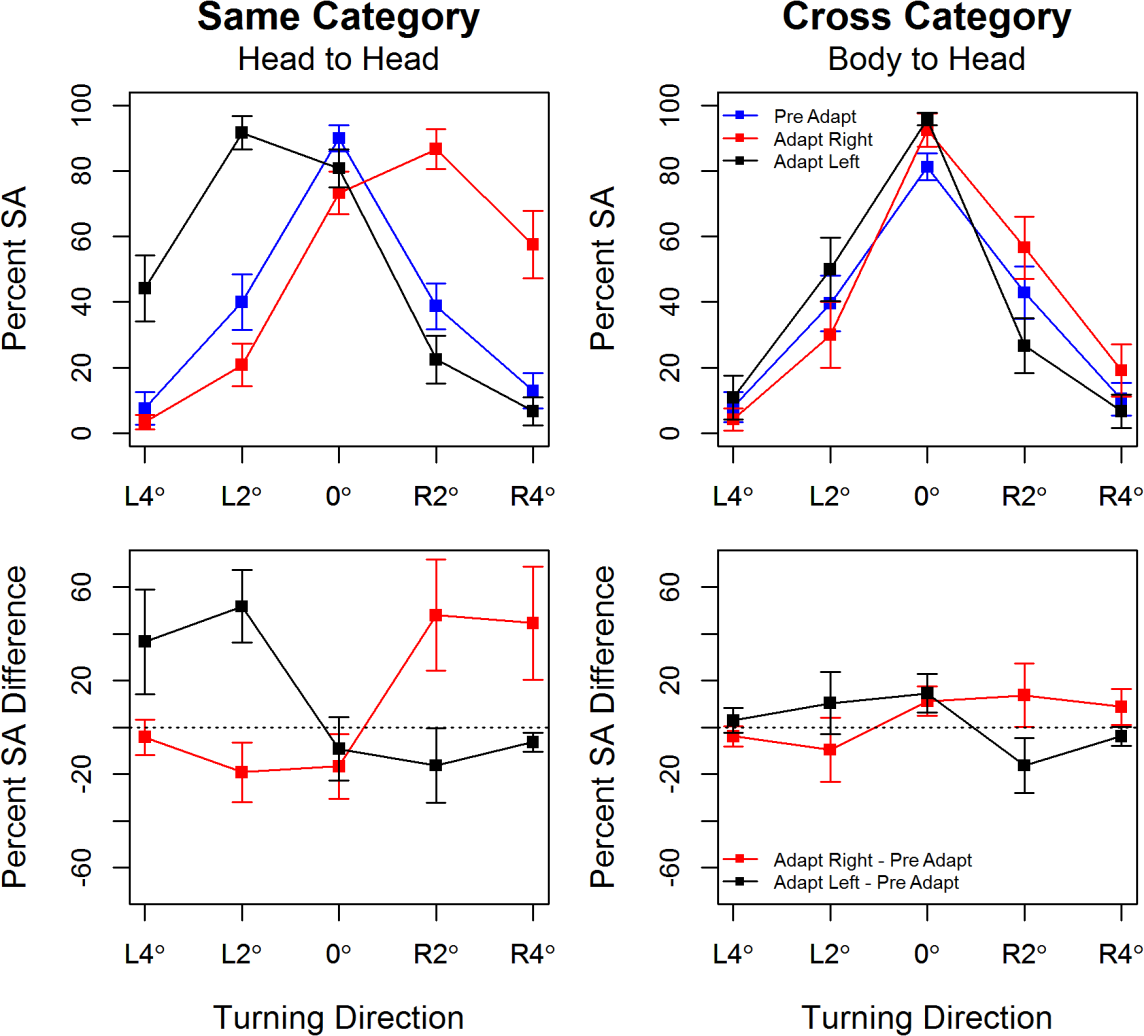
Top Panel: Percentage straight ahead responses by test head orientation in Experiment 2. Error bars show +/1 S.E.M. Bottom Panel: Mean difference in the percentage of straight ahead responses pre- and post-adaptation by test body orientation. Error bars show 95% confidence intervals around the mean.

Using a range of smaller head orientations, namely 4° and 2° left, 0°, 2° and 4° right, had the desired effect with participants perceiving heads oriented at 2° as pointing straight ahead on ~40% of baseline trials. The pattern of responses after head-to-head adaptation is very similar to that seen for body-to-body adaptation in Experiment 1 (Fig 3 and Table 2). Specifically, after adaptation to heads oriented to the right (*left*) the neutral stimulus appears to face in the opposite direction. Secondly, heads that are physically oriented in the same direction as the adaptor are more often perceived as facing straight ahead after adaptation, so that there is a clear shift in the neutral point. Finally, tuning is sharpened for heads oriented in the opposite direction to the adapting stimulus. While this pattern of adaptation is evidence in the cross-category (body to head) condition, the effects are much weaker and the pre-adaptation and post-adaptation curves in Fig 3 are largely overlapping.

**Table 2:** Response Change Post Adaptation Compared to Baseline.

**Experiment 2**Same Adaptation (Head to Head)				
	*Adapt Right*: *Baseline*		*Adapt Left*: *Baseline*	
**Test Body**	**Response Change**	**Percent SA Change [95% CI]**	**Response Change**	**Percent SA Change [95% CI]**
4° L	↓SA, ↑L	-4.17 [-11.69:3.36]	↑SA, ↓L	36.67 [14.31:59.02] *
2° L	↓SA, ↑L	-19.17 [-31.86:-6.47] *	↑SA, ↓L	51.67 [36.14:67.19] *
0°	↓SA, ↑L	-16.67 [-30.50:-2.83] *	↓SA, ↑R	-9.17 [-22.77:4.44]
2° R	↑SA, ↓R	47.92 [24.13: 71.71] *	↓SA, ↑R	-16.25 [-32.29:-0.21] *
4° R	↑SA, ↓R	44.58 [20.45:68.72] *	↓SA, ↑R	-6.25 [-10.34:-2.16]
**Experiment 2**Cross Adaptation (Body to Head)				
	*Adapt Right*: *Baseline*		*Adapt Left*: *Baseline*	
**Test Body**	**Response Change**	**Percent SA Change [95% CI]**	**Response Change**	**Percent SA Change [95% CI]**
4° L	↓SA, ↑L	-3.75 [-8.06:0.56]	↑SA, ↓L	2.92 [-2.41:8.24]
2° L	↓SA, ↑L	-9.58 [-23.36:4.20]	↑SA, ↓L	10.42 [-2.82:23.65]
0°	↑SA, ↓R	11.25 [4.88:17.62]	↑SA, ↓L	14.58 [6.29:22.87]
2° R	↑SA, ↓R	13.75 [0.26: 27.24] *	↓SA, ↑R	-16.25 [-27.99:-4.51] *
4° R	↑SA, ↓R	8.75 [1.07:16.43]	↓SA, ↑R	-3.75 [-7.84: 0.34]

Note. SA = Straight Ahead responses; L = Left responses; R = Right responses.

* Substantial effect in the expected direction, and the 95% CIs do not include zero

The omnibus test showed a significant Orientation*Adaptation*Condition interaction (*p*~0). ANOVA for same-category adaptation showed a significant Orientation*Adaptation interaction, F(8,88) = 31.33, *p* ~ 0.00, *η2G* = 0.50, as did ANOVA for cross-category adaptation, F(8,88) = 9.64, *p* ~ 0.00, *η2G* = 0.09. These interactions are explored in Table 2 and the lower panel of Fig 3. For head-to-head adaptation the effect sizes in the table cells with an asterisk run between a low of 15.42% and a high of 59.58% after rightward adaptation at 2°L, 0°, 2°R and 4°R, and after leftward adaptation at 2°R, 2°L and 4°L. The effect sizes are smaller at 0°, -16.67% after rightward and -9.17% (with the 95% CI including zero) after leftward adaptation, when compared to the effects found for body-body adaptation in Experiment 1. Again, perception of forward facing heads is particularly robust.

In the cross-category condition, the evidence for adaptation is much weaker with smaller effect sizes and fewer 95% CIs that do not include zero (Fig 3, Table 2). There is a small and unexpected *increase* in the percentage of straight ahead responses at 0° following both rightward and leftward adaptation; viewing extremely oriented body stimuli appears to strengthen the perception of forward facing heads. In summary, Experiment 2 shows clear evidence of head to head adaptation but little or no evidence of body head adaptation.

## Discussion

This study presents the first evidence of *cross-category* adaptation to head and body orientation by way of a novel direction-specific perceptual aftereffect. Adapting to images of extremely oriented heads produces a perceptual bias in judging the turning direction of subsequently presented bodies. In contrast, little to no change in the judgment of head orientation occurs when participants adapt to extremely oriented bodies. The unidirectional nature of the aftereffect suggests that head orientation modulates the perception of body orientation but that information about body orientation does not inhibit the perception of head orientation, a finding consistent with the idea that cues from the human body signaling social attention are combined in a hierarchical fashion whereby cues to social attention from the head may override those of the body [10].

As expected and in line with previous research [6,8,9] adapting to side views of human bodies and heads biases the perceived direction of subsequently viewed bodies and heads respectively, and these strong viewpoint aftereffects provide support for the existence of view-selective neurons in the visual system. The aftereffects are unlikely to reflect low-level adaptation as the adaptor and test stimuli varied in shape, in size and in gender and identity. Secondly, the relatively short presentation of test stimuli (300ms) suggests that the aftereffects do not follow from an extended cognitive analysis of individual face or body features but is based on fast global processing as, like faces, bodies are processed configurally [26]. Finally, in the specific case of *cross-category* adaptation, the adapting and test stimuli did not share the same configuration of local features. Therefore, an interpretation of these results as reflecting high-level adaptation to head and body orientation is warranted, with the *cross-adaptation* effects having important implications for understanding the neural coding of head and body viewpoint.

Our results also point to a particularly robust representation of forward facing heads. First, participants’ judgments that a head is oriented ‘straight ahead’ are less susceptible to same-category adaptation (Experiment 2) than are bodies (Experiment 1) so that there is a much less obvious shift in the neutral point for head to head than for body to body adaptation. Secondly, adaptation to extremely oriented bodies in Experiment 2 strengthens rather than weakens the perception of forward facing heads as facing ‘straight ahead’. As our heads were rendered with eyes closed, this is unlikely to reflect the recently reported expectation bias that gaze is usually directed at the observer [27].

Previous research on the viewpoint aftereffect [6] found no cross adaptation between images of human heads and of other objects and concluded that neurons selective for viewpoint are also selective for object categories. However, the stimulus categories used in [6] were biological and non-biological. In contrast, we used heads and bodies that are two categories of the one biological form. While cross adaptation between different viewpoints of these two social signals has not been previously examined, cross adaptation aftereffects have been reported for faces and bodies along the higher order dimension of gender. Interestingly, unlike our viewpoint aftereffects, which are directional in nature, the gender aftereffects transfer both ways. Continuous viewing of male or female bodies [18] or heads [28] leads to the opposite gender bias in the perception of subsequently presented faces or bodies respectively. Coupled with reports that gender aftereffects can also be induced by continuous viewing of gender-specific objects (e.g., lipstick, shoes, items of clothing etc.), it is likely that these effects evidence neuronal populations tuned to the higher order, learned, concept of gender [29].

In the case of the viewpoint aftereffects reported here, adaptation likely occurs at neuronal sites where information about the human face and body are integrated. Single cell recordings from the anterior region of macaque STS reveal whole body selective cells tuned to both orientation and size [30]. While *cross-category* viewpoint invariant responses–via response pooling across orientation tuned cells–may be necessary for object recognition, the authors argue that the retention of information about viewpoint is crucial for interpreting social signals. Indeed, neuroimaging research points to human anterior STS in coding both head orientation and eye-gaze direction [12,31]. The fusiform gyrus is also implicated in processing head [12] and body facing direction [32].

As described above, behavioural studies examining cue integration provide conflicting accounts as to how cues to social attention are combined, with different experimental paradigms supporting different accounts. For example, both [5] and [12] use a Stroop-like task and report that cues from the head and from the eyes influence each other in a bidirectional manner. In Langton [5] participants were shown images of a head turned to the right or left with the eyes gazing in the same or opposite direction. In separate blocks they made speeded responses to indicate the direction of eye gaze or the turning direction of the head whilst ignoring the second cue. This study reports symmetrical interference effects such that judgements of both eye gaze and head orientation were slowed when the to be ignored cue was incongruent with the cue participants were attending to. This suggest that social cues to attention are processed in parallel and later combined in an additive manner such that one cue can inhibit the other and *vice versa*.

In contrast, research using the modified Posner paradigm supports a hierarchical model of cue integration. In Hietanen [3,4] participants made speeded responses to indicate whether a peripheral target appeared to the right or left of a central fixation with each response trial preceded by a brief, centrally presented directional cue. When heads were used as the directional cue [3] they only served to speed up response times when the eye gaze and head turning direction were in different directions. Specifically, a frontally facing head with eye gaze averted to the right or left led to faster responses when eye gaze direction was predictive of target location. In contrast, heads which were turned to the right or left with eyes gazing back to the participant did not speed up response times when head turning predicted target location. And somewhat surprisingly, when both head turning and eye gaze were in the same direction and predictive of target location, they did not serve to speed response times. Interestingly, this identical stimulus (head turned to the right or left with eyes gazing in the same direction) did lead to significantly faster response times when combined with a body which was facing the observer [4].

Together these two studies suggest, first, that social cues to attention are judged with reference to the perspective of the person we are looking at. When observing another person, averted eyes (relative to their head orientation) or a turned head (relative to their body orientation) are strong cues to a shift in that person’s attention and they draw our attention in the same direction. Secondly, these results suggest that cues are combined hierarchically such that eye gaze is referenced to head orientation and head orientation is referenced to body orientation. A recent study using a novel ‘representational momentum’ paradigm, in which participants judged the endpoint of a just viewed rotating head also points to the strong influence of eye-gaze information on the perception of head orientation in a dynamic task [33].

The unidirectional nature of the cross adaptation aftereffects observed in the current study supports the integrative hierarchical model of cue combination. To put our findings in the context of other research in this area, we note, first, that they are consistent with predictions based on the seminal single cell studies of Perrett and colleagues [10]. As adaptation offers a non-invasive and direct way to study the neural coding of visual information, it stands as a straightforward test of the DAD model of cue integration. Secondly, although our study involves an overt direction discrimination task our results are consistent with findings from the spatial cueing research [3,4], which points to the primacy of eye-gaze over head orientation and of head over body orientation when these are placed in conflict. It could be argued, as suggested by a reviewer, that our findings measure cue integration at the level of perceptual processing rather than at the level of attentional cueing. In response we note our assumption, in linking our findings to those from the spatial cueing research, that the attentional system draws directly on underlying perceptual mechanisms. This issue has been addressed recently for the case of single cues to social attention [34]. where it was shown that the effectiveness of eye gaze as a directional cue may be diminished as a direct result of perceptual adaptation to eye gaze direction. This paradigm, which combines perceptual adaptation with spatial cueing offers a way to further explore the links between perceptual and attentional mechanisms and to addresses the important question as to the role of social perception in social cognition, e.g., deficits in the following of eye gaze has been posited to underlie deficits in joint attention and social cognition in autism [35].

In summary, our findings show cross adaptation between two cues integral to social attention, head and body orientation. The asymmetrical nature of the aftereffects we observe has important implications for understanding how these cues integrate in the visual system and support the hierarchical model of cue combination.

## Supporting Information

S1 Fig Top Panel: Percentage straight ahead responses by test head orientation in Experiment 2(a). Error bars show +/1 S.E.M. Bottom Panel: Mean difference in the percentage of straight ahead responses pre- and post-adaptation by test body orientation. Error bars show 95% confidence intervals around the mean.
(TIFF)
