# Supplementary figures and images for: Heads First: Visual Aftereffects Reveal Hierarchical Integration of Cues to Social Attention
Source: PLoS One. 2015 Sep 11;10(9):e0135742. doi: 10.1371/journal.pone.0135742 (PMC4567288; doi:10.1371/journal.pone.0135742)

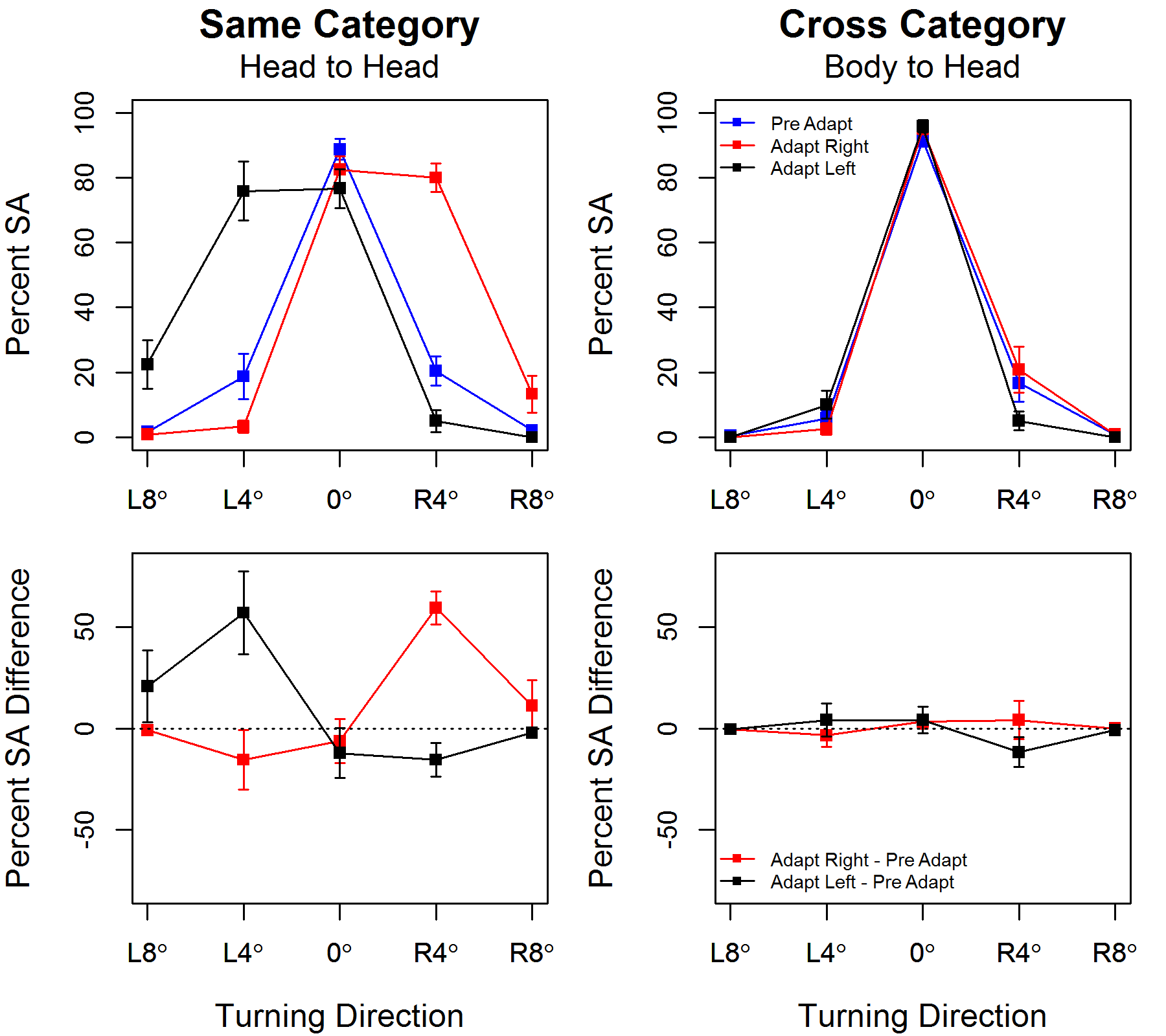

Supplement: S1 Fig — Error bars show +/1 S.E.M. Bottom Panel: Mean difference in the percentage of straight ahead responses pre- and post-adaptation by test body orientation. Error bars show 95% confidence intervals around the mean. (TIFF) [file pone.0135742.s001.tiff]
